# Narrowing the Patient–Physician Gap Based on Self-Reporting and Monthly Hepatologist Feedback for Patients With Alcohol-Related Liver Disease: Interventional Pilot Study Using a Journaling Smartphone App

**DOI:** 10.2196/44762

**Published:** 2023-12-19

**Authors:** Noriyo Yamashiki, Kyoko Kawabata, Miki Murata, Shunichiro Ikeda, Takako Fujimaki, Kanehiko Suwa, Toshihito Seki, Eiji Aramaki, Makoto Naganuma

**Affiliations:** 1 Department of Gastroenterology and Hepatology Kansai Medical University Medical Center Osaka Japan; 2 The Third Department of Internal Medicine Division of Gastroenterology and Hepatology Kansai Medical University Osaka Japan; 3 Graduate School of Science and Technology Nara Institute of Science and Technology Ikoma Japan; 4 Department of Psychiatry Kansai Medical University Osaka Japan

**Keywords:** alcohol-related liver disease, alcohol use disorder, alcoholism, smartphone, mobile health

## Abstract

**Background:**

Screening and intervention for alcohol use disorders (AUDs) are recommended to improve the prognosis of patients with alcohol-related liver disease (ALD). Most patients’ smartphone app diaries record drinking behavior for self-monitoring. A smartphone app can be expected to also be helpful for physicians because it can provide rich patient information to hepatologists, leading to suitable feedback. We conducted this prospective pilot study to assess the use of a smartphone app as a journaling tool and as a self-report–based feedback source for patients with ALD.

**Objective:**

The aims of this study were assessment of whether journaling (self-report) and self-report–based feedback can help patients maintain abstinence and improve liver function data.

**Methods:**

This pilot study used a newly developed smartphone journaling app for patients, with input data that physicians can review. After patients with ALD were screened for harmful alcohol use, some were invited to use the smartphone journaling app for 8 weeks. Their self-reported alcohol intake, symptoms, and laboratory data were recorded at entry, week 4, and week 8. Biomarkers for alcohol use included gamma glutamyl transferase (GGT), percentage of carbohydrate-deficient transferrin to transferrin (%CDT), and GGT-CDT (GGT-CDT= 0.8 × ln[GGT] + 1.3 × ln[%CDT]). At each visit, their recorded data were reviewed by a hepatologist to evaluate changes in alcohol consumption and laboratory data. The relation between those outcomes and app usage was also investigated.

**Results:**

Of 14 patients agreeing to participate, 10 completed an 8-week follow-up, with diary input rates between 44% and 100% of the expected days. Of the 14 patients, 2 withdrew from clinical follow-up, and 2 additional patients never used the smartphone journaling app. Using the physician’s view, a treating hepatologist gave feedback via comments to patients at each visit. Mean self-reported alcohol consumption dropped from baseline (100, SD 70 g) to week 4 (13, SD 25 g; *P*=.002) and remained lower at week 8 (13, SD 23 g; *P*=.007). During the study, 5 patients reported complete abstinence. No significant changes were found in mean GGT and mean %CDT alone, but the mean GGT-CDT combination dropped significantly from entry (5.2, SD 1.2) to the week 4 visit (4.8, SD 1.1; *P*=.02) and at week 8 (4.8, SD 1.0; *P*=.01). During the study period, decreases in mean total bilirubin (3.0, SD 2.4 mg/dL to 2.4, SD 1.9 mg/dL; *P*=.01) and increases in mean serum albumin (3.0, SD 0.9 g/dL to 3.3, SD 0.8 g/dL; *P*=.009) were recorded.

**Conclusions:**

These pilot study findings revealed that a short-term intervention with a smartphone journaling app used by both patients and treatment-administering hepatologists was associated with reduced drinking and improved liver function.

**Trial Registration:**

UMIN CTR UMIN000045285; http://tinyurl.com/yvvk38tj

## Introduction

### Background

Alcohol-related liver disease (ALD), including cirrhosis-related death and hepatocellular carcinoma, is a leading cause of liver-related death [1,2]. In the Asia-Pacific region, alcohol has become the third most common cause of liver-related deaths after hepatitis B and hepatitis C [3]. Consequently, liver disease etiology has been changing from viral hepatitis to non-viral liver diseases [4-7].

As one part of treatment strategies, abstinence from alcohol is strongly recommended for patients with alcoholic cirrhosis or alcoholic hepatitis [8]. In primary care settings, “screening and brief intervention” are effective to reduce drinking in the general population [9]. Recent guidelines have also recommended this screening and brief intervention approach for patients with ALD [8,10]. An Alcohol Use Disorders Inventory Test (AUDIT) score greater than 8 is predictive of harmful or hazardous alcohol use. A score greater than 20 is suggestive of moderate-to-severe alcohol use disorder (AUD) [8,10]. Alcohol screening questionnaires including CAGE [11,12] and the AUDIT-C (3 questions related to alcohol consumption from the full questionnaire) [13-15] in an outpatient setting are useful to predict subsequent hospitalization for gastrointestinal conditions [16]. Moreover, the usefulness of the AUDIT-C to identify risks of alcohol relapse after liver transplantation has also been shown [17].

For patients with moderate-to-severe AUD, a brief intervention is not sufficient. Referral to treatment for AUD by an addiction specialist is ideal [8,10]. However, among high-risk drinkers, the proportion of patients receiving treatment for liver dysfunction or alcoholism is low. A survey revealed that only 0.8% of risky drinkers wish to stop drinking and that only 5.1% of risky drinkers are undergoing treatment for liver dysfunction [18]. These reports suggest that many patients with ALD and AUD refuse to reduce drinking or to accept addiction counseling. Considering this background, the key issue with AUD treatment for patients with ALD is reduction of patients’ drinking by hepatologists, even without the support of addiction specialists.

Digital tools including smartphone apps might be used as feedback and planning support, educational tools, or other support in order to improve health care [19].

### Feedback and Planning Support

Feedback is a core service supported by most systems. Popular feedback items provided to users are cost and calorie equivalents to a person’s alcoholic beverage consumption, as well as drinking-related health risks [19,20]. Scott and colleagues [21] reported a project to assess granular self-monitoring feedback using ecological momentary assessments and immediate recovery support through ecological momentary interventions.

### Education and Coaching

Educational support provides knowledge related to diseases (alcohol addiction) and risks associated with alcohol. It enables the encouragement of self-motivated alcohol reduction. Drinkaware [22] proposes the following educational factors: assessment and feedback, high-risk places for drinking, choosing and using supportive people for change, cravings and their management, problem-solving skills, communication, refusal to drink skills, and fun nondrinking activities.

### Emergency Support

Emergency support is useful in managing expected or out-of-control situations. The Addiction-Comprehensive Health Enhancement Support System (A-CHESS) [23] provides emergency support called the *panic button*. If the patient presses the panic button, then the system sends an emergency message to 2 contact persons who have been prepared in advance.

### Effectiveness of Existing Apps

Most apps have successfully demonstrated effectiveness. One key issue is safety. For example, Glowacki et al [24] reported that a mismatched text message sometimes exacerbates unhealthy behavior.

Another issue is that these approaches have been examined in settings of alcohol dependence but not in settings of ALD. Reported smartphone apps are used mainly by patients [20-26]; physicians do not use them.

A smartphone app is presumably able to provide rich patient information to hepatologists, leading to effective feedback. To assess patient information, a physician (hepatologist) can review the following 2 types of information from a patient's input: (1) alcohol consumption that indicates the patient's drinking status and (2) a patient narrative that can imply the patient's motivation. Using that information, hepatologists can give suitable feedback to patients.

### Objectives

The objectives of this study were to assess the use of a smartphone app as a journaling tool and a source of feedback based on self-report for patients with ALD, specifically its effects on (1) abstinence from alcohol and (2) improvement in biomarkers of alcohol use and liver function tests.

## Methods

### Patients

The target hospital was a main hospital in the Osaka area of Japan. Approximately 200 patients with liver disease are seen in the outpatient department each week, including about 10 cases referred from general practitioners. During August 2021 to May 2022, we recruited patients with ALD who were seen either in the hospital or an outpatient clinic. When the patient’s liver disease was regarded as caused by harmful alcohol intake, patients were given educational booklets that included information about health risks and harmful alcohol use. Abstinence from alcohol was recommended by hepatologists (NY, MM, KS), especially for patients with liver cirrhosis or hepatocellular carcinoma. This was advice with no implied nor actual penalty. When AUD was suspected based on the AUDIT score [13], psychiatric outpatient care was provided with consent, but no recovery program was routinely provided at our hospital. When an ALD patient was admitted to our hospital for any liver-related condition, a psychiatric liaison team round was scheduled by agreement.

**Figure 1:**
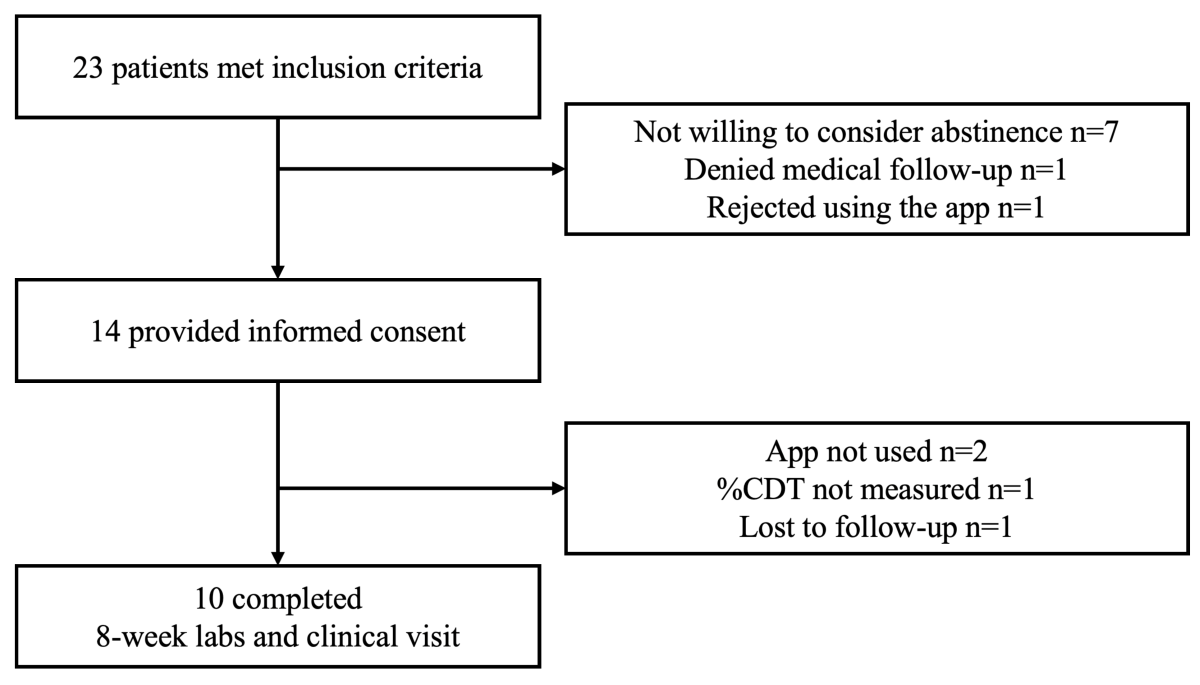
Patient flow. %CDT: percentage of carbohydrate-deficient transferrin to transferrin.

No patient who had been abstinent for more than 1 year or who had refused to reduce or abstain from alcohol intake was invited to participate in the study. During the period, 23 patients were invited, 14 of whom provided written informed consent. The patient flow is portrayed in Figure 1.

### Data Collection and Follow-Up

Outpatient appointments were set at week 4, week 8, and week 24. However, changes to appointments were allowed 1 week before and after the date that had been initially set.

Patient physical data and clinical characteristics were obtained at the time of entry into the study. Laboratory data, including aspartate aminotransferase, alanine aminotransferase, gamma glutamyl transferase (GGT), total bilirubin, albumin, blood cell count, prothrombin international normalized ratio, and serum percentage of carbohydrate-deficient transferrin to transferrin (%CDT) level, were measured at baseline, week 4, week 8 and week 24. The level of serum %CDT was analyzed using direct immunonephelometric assay (N Latex CDT; Siemens Healthcare Diagnostics). A combined index based on GGT and CDT measurements (GGT-CDT) has been shown to improve sensitivity to detect heavy drinkers and was calculated as [27] GGT-CDT = 0.8 × ln(GGT) + 1.3 × ln(%CDT).

### Feedback From Hepatologists

At each follow-up visit (week 4 and week 8), the treating hepatologist (NY, MM, and KS) provided self-report–based feedback in addition to regular outpatient practice. The interventions in this study are presented in Table 1. The hepatologist referred to the “physician’s view” to review whether drinking status was reported, how much ethanol was consumed on average, and what type of input was made. An example of using a physician’s view in an outpatient setting is the following: A patient and a clinician viewed the graph of alcohol consumption together, thereby enabling the patient to confirm the amount and frequency of alcohol consumption. However, we used no specific feedback protocol. The feedback manner differed among hepatologists because no special training for behavioral health was executed.

**Table 1:** Intervention in this study in comparison with regular outpatient practice.

Intervention	This study	Regular outpatient practice
Attending physician	Hepatologist	Hepatologist
Outpatient frequency	Monthly (entry and weeks 4 and 8 are mandatory)	Every 1 month to 3 months
Self-treatment for alcohol use	Educational booklets about alcohol risks plus journaling (smartphone app)	Educational booklets about alcohol risks
Outpatient treatment for alcohol use (available information)	Feedback (liver function tests plus alcohol consumption plus patient report)	Feedback according to laboratory results for liver function tests
Referral to psychiatrists	In agreement	In agreement
Hospitalization/inpatient service	Hospitalization to the gastrointestinal (GI) unit as indicated/liaison team round in agreement	Hospitalization to GI unit as indicated/liaison team round in agreement
Addiction team counseling	Referral with consent	Referral with consent

### Smartphone App Development

There were 2 developed systems: (1) a client smartphone journaling app for patients and (2) a server app for physicians’ use.

#### Smartphone App

**Figure 2:**
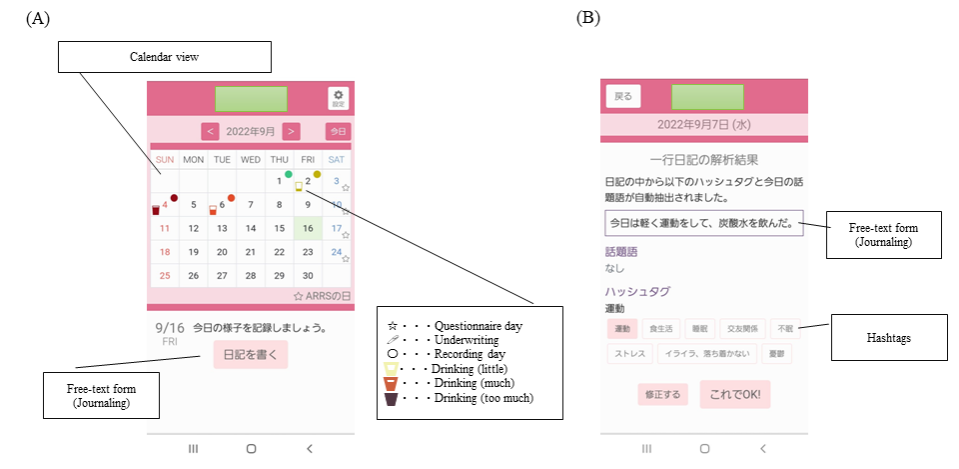
Smartphone app (A) month view and (B) day view.

A web-based smartphone journaling app was provided. Each patient reported their drinking status and an associated narrative. The drinking status data were converted automatically into ethanol amounts. A screenshot of the app is presented in Figure 2. To record in the diary, text input is required (Figure 2B). Patients can monitor the drinking amount as colored icons (ie, green denoting no alcohol consumption, and red denoting drinking more than 60 g of ethanol). During this study, patients were required to use this smartphone journaling app from the entry day until the clinic visit appointment at week 8. Patient comments on the smartphone app were collected via a questionnaire at week 8. After 8 weeks, the app was used on a voluntary basis.

#### Server App

**Figure 3:**
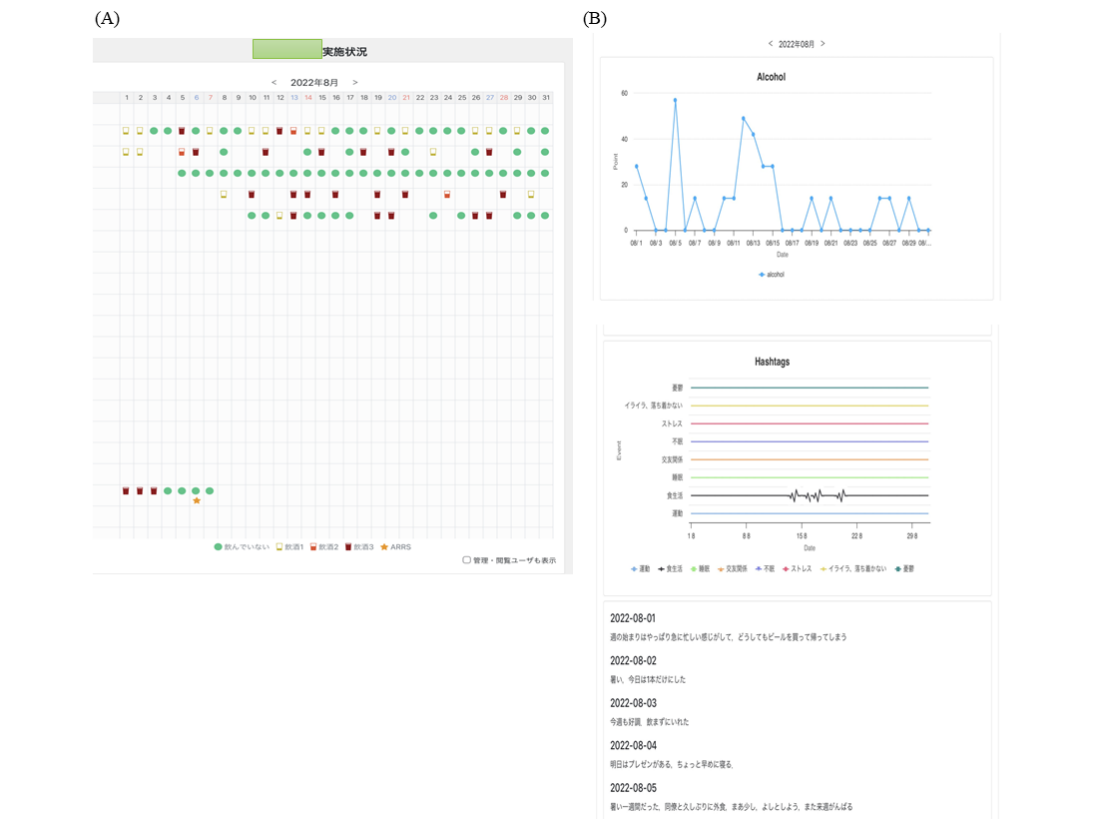
Physician interface: (A) overview and (B) detailed view.

At the outpatient clinic, the treating doctor referred to the doctor’s view of the patient input (Figure 3). The amount of daily alcohol intake is shown as a line graph of the ethanol equivalent (Figure 3A). All diary inputs are also shown (Figure 3B).

Using the server app, the percentage of diary inputs and the average alcohol intake were calculated as:

Diary input rate (%) = (number of days with a diary recording/number of days from entry through week 8) × 100

Daily ethanol consumption (g/day) = sum of daily ethanol use (g)/number of days with a diary recording during the expected period

Lags between the date of the smartphone journaling app and the date and time of the response were calculated using server data. If a day’s diary data were entered on the following day, then the input lag was designated as 1 day. We designated this gap as the input time lag.

### Statistical Analyses

Normally or near-normally distributed variables are reported as means and SDs. Equality between groups was compared using the Student *t* test. The results obtained before and after the intervention were compared using paired *t* tests. Non-normally distributed continuous data are reported as medians and ranges. They were compared using Wilcoxon rank-sum tests. Categorical variables were compared using chi-square tests or Fisher exact tests when appropriate. Correlations between self-reported average daily ethanol consumption and biomarkers of alcohol use were analyzed using Pearson correlation coefficient tests. All analyses were conducted using software (STATA ver. 15; Stata Corp). *P* values less than .05 were considered significant.

### Ethical Considerations

This study was approved by the Kansai Medical University Center for Ethical Review (No. 2020252) and was conducted in compliance with the Declaration of Helsinki.

## Results

Baseline patient information and clinical characteristics of the 14 patients are presented in Table 2: 8 were male, one-half (7/14, 50%) resided alone, 6 were unemployed, 5 were consulting a psychiatrist for various reasons, and 3 had been previously diagnosed with AUD. None of the recruited patients agreed to receive a new referral to the addiction team during this 8-week period. Alcohol was used daily by 12 (86%) of the 14 patients, while 2 were non-daily drinkers. The average ethanol intake was 97 (SD 61) g daily. The mean AUDIT score at entry was 20 (SD 8). Of the 14 patients, 10 (71%) were diagnosed with cirrhosis. The patients included in this study were not transplant candidates.

**Table 2:** Baseline patient information and clinical characteristics (n=14).

Factor		Values
Age (years), mean (SD)		51.3 (10.5)
Male gender, n (%)		8 (57)
Race/ethnicity (Asian), n (%)		14 (100)
Body mass index (kg/m^2^), mean (SD)		24.9 (4.1)
**Smoking, n (%)**		
	Current	10 (71)
	Past	2 (14)
	None	2 (14)
Living alone, n (%)		7 (50)
Unemployed, n (%)		6 (43)
**Psychiatric comorbidity,** **n** **(%)**		
	AUD^a^, depression, and anorexia	1 (7)
	AUD and depression	1 (7)
	AUD	1 (7)
	Anorexia	1 (7)
	Panic disorder	1 (7)
	None	9 (64)
**Medical comorbidity,** **n** **(%)**		
	Hypertension	1 (7)
	Diabetes	1 (7)
	Posttreatment hepatitis C	1 (7)
	Chronic pancreatitis	1 (7)
Duration of drinking (years), mean (SD)		31 (10)
Daily ethanol intake (g), mean (SD)		97 (61)
AUDIT^b^ score at entry, mean (SD)		20 (8)
**AUDIT score at entry, n (%)**		
	≥8	13 (93)
	≥20	8 (57)
Aspartate aminotransferase (AST; U/L), mean (SD)		71 (43)
Alanine aminotransferase (ALT; U/L), mean (SD)		47 (46)
Gamma glutamyl transferase (GGT; U/L), mean (SD)		359 (448)
Total bilirubin (mg/dL), mean (SD)		2.8 (2.3)
Albumin (g/dL), mean (SD)		3.0 (0.9)
Platelets (10 × 10^3^/μL), mean (SD)		15.7 (10.0)
White blood cells (/μL), mean (SD)		5500 (1687)
INR^c^, mean (SD)		1.37 (0.42)
%CDT^d^ (%), mean (SD)		2.60 (1.01)
Cirrhosis, n (%)		10 (71)
Child-Pugh score, median (range)		8 (5-13)
Child class B or C, n (%)		8 (57)

^a^AUD: alcohol use disorder.

^b^AUDIT: Alcohol Use Disorders Inventory Test.

^c^INR: international normalized ratio.

^d^CDT: carbohydrate-deficient transferrin.

Recording data are presented in Table 3. Overall, 10 of 14 (71%) patients completed 8 weeks of follow-up and laboratory tests (cases 1-10). Of the 14 patients, 2 were lost to follow-up (cases 12, 14), 2 never filled in the data (cases 13, 14), and 1 filled in only the last one-half of the data (case 11) in the smartphone app. Among the 10 patients who completed 8 weeks of follow-up, their 8-week visit actually occurred between day 56 and day 70 from entry for 7 cases, but the follow-up was delayed up to day 119 for 3 cases due to patient request. Of the 10 patients, 5 patients were able to use the smartphone journaling app continually (input rate of more than 70%) and were able to abstain from alcohol completely. However, 5 patients continued to use the smartphone journaling app (input rate of 44%-100%) but were unable to abstain from alcohol completely. Of these 5 patients, 4 had an intake that exceeded 20 g of pure alcohol on 80% to 100% of the days on which they drank (data not shown in the table).

**Table 3:** Summary of recorded data and input time lag.

Case number	Input rate			All periods			Entry to week 4				Week 4 to week 8		
	Expected recording days, n	Days with input, n (%)	Days abstinent, n (%)		Days drinking, n (%)	Days with input, n (%)		Input time lag (days), mean (SD)	Alcohol intake (g), mean (SD)	Days with input, n (%)		Input time lag (days), mean (SD)	Alcohol intake (g), mean (SD)
1	67	52 (77)	52 (77)		0	39 (100)		0.6 (0.8)	0	13 (46)		0	0
2	68	61 (89)	61 (89)		0	34 (82)		1.1 (1.1)	0	27 (100)		2.7 (1.8)	0
3	63	57 (90)	57 (90)		0	33 (94)		0.1 (0.3)	0	24 (85)		0.3 (0.4)	0
4	65	63 (96)	63 (96)		0	35 (94)		0.1 (0.3)	0	28 (100)		0.0 (0.1)	0
5	82	82 (100)	82 (100)		0	28 (100)		1.6 (0.9)	0	35 (100)		4.2 (3.8)	0
6	63	28 (44)	3 (4)		25 (39)	28 (100)		3 (2)	51 (28)	0		N/A^a^	N/A
7	77	39 (50)	31 (40)		8 (10)	4 (9)		9.7 (14.5)	7 (12)	35 (100)		12.7 (9.0)	7 (17)
8	70	61 (87)	13 (18)		48 (68)	34 (97)		1.1 (1.1)	42 (23)	27 (77)		6.1 (4.5)	40 (22)
9	119	119 (100)	15 (12)		104 (87)	42 (100)		8.2 (7.3)	76 (23)	77 (100)		18.5 (11.3)	68 (30)
10	68	52 (76)	29 (42)		23 (33)	34 (97)		0.1 (0.3)	4 (6)	27 (81)		0.8 (0.6)	18 (27)
11	85	29 (34)	29 (34)		0	1 (1)		0	0	28 (100)		8.5 (6.8)	0
12	28	8 (28)	2 (7)		6 (21)	8 (28)		1.8 (1.2)	46 (35)	0		N/A	N/A
13	0	0	—^b^		—	—		—	—	—		—	—
14	0	0	—		—	—		—	—	—		—	—

^a^N/A: not applicable.

^b^No data.

**Figure 4:**
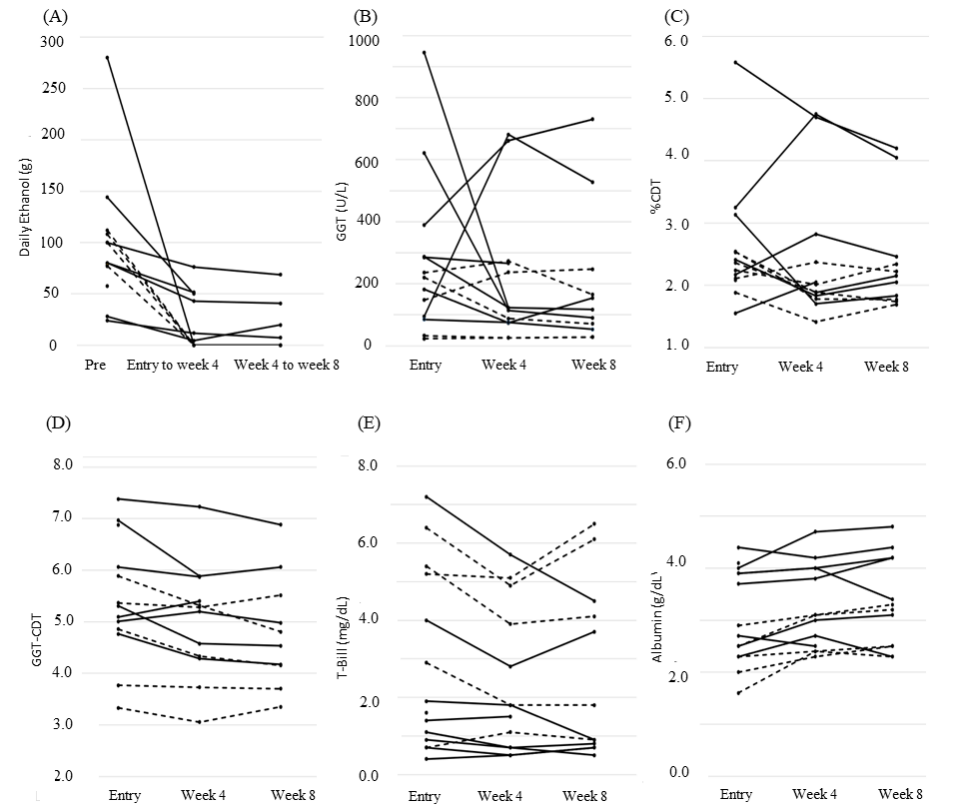
(A) Self-reported average daily alcohol use as grams of ethanol; changes in (B) gamma glutamyl transferase (GGT), (C) percentage of carbohydrate-deficient transferrin to transferrin (%CDT), (D) GGT-CDT, (E) total bilirubin (T-Bili), and (F) albumin. Solid lines indicate cases who did not abstain from drinking, while dashed lines indicate cases who completely abstained.

Changes in the self-reported average daily ethanol intake according to the diary inputs are shown in Figure 4A. For the 10 patients who filled in their diary during the study period, the average ethanol intake decreased significantly from baseline (100, SD 70 g) to week 4 (13, SD 25 g; *P*=.002) and from baseline to week 8 (13, SD 23 g; *P*=.007), but no change was apparent from week 4 through week 8 (*P*=.94).

No significant correlation was found between daily ethanol consumption and GGT (*r*=0.23, *P*=.19), %CDT (*r*=0.25, *P*=.16), or GGT-CDT (*r*=0.24, *P*=.18).

Changes in laboratory data are presented in Figures 4B-4F. No significant changes were found for mean GGT and mean %CDT alone, but the mean GGT-CDT combination dropped significantly from entry (5.2, SD 1.2) to the week 4 visit (4.8, SD 1.1; *P*=.02) and the week 8 visit (4.8, SD 1.0; *P*=.01; Figures 4B, 4C, and 4D). The total bilirubin level dropped significantly from entry (3.0, SD 2.4 mg/dL) to the week 4 visit (2.4, SD 1.9 mg/dL; *P*=.01), but no additional change was observed from week 4 through week 8 (2.6, SD 2.3 mg/dL; *P*=.53; Figure 4E). The mean serum albumin level increased significantly from entry (3.0, SD 0.9 g) to week 4 (3.3, SD 0.8 g/dL; *P*=.009). The same tendency was maintained until week 8 in cases of complete abstinence (Figure 4F).

The relation between the input time lags and amount of alcohol consumed is shown in Table 3. Irrespective of the amount of alcohol consumed, the input time lags remained small until week 4. Subsequently, the number of days that the input lagged increased gradually. Respondents had a tendency to input missing amounts immediately before their week 8 follow-up visit (Multimedia Appendix 1). Participants who were not completely abstinent and drank heavily tended to have larger input time lags than those who were completely abstinent. The input time lags were not large for those who were completely abstinent.

We also collected patient comments about the smartphone app via a questionnaire (Multimedia Appendix 2). The results indicated that the “app usability” and “ease of viewing the app screen” were highly rated: 1 patient aged in his 60s commented that the screen was difficult to read. Three patients reported that using the app helped them abstain from drinking more or less, 2 patients said they wanted some kind of feedback function, and 3 patients expressed negative opinions about keeping a diary. Additional comments are presented in Multimedia Appendix 2. 

## Discussion

### Principal Findings and Comparison With Earlier Work

This pilot study evaluated the use of a smartphone journaling app by patients with ALD. About 60% of candidate patients were evaluated in this study. Of them, 71% completed 8 weeks of journaling. One-half of the patients who continued diary input achieved 8 weeks of complete abstinence.

With ALD on the rise, efforts for harm reduction and alcohol consumption reduction are attracting attention [28]. For patients with both ALD and AUD, screening and brief interventions and referral to treatment are useful. Subsequent pharmacotherapy and psychiatric intervention such as cognitive therapy and motivation enhancement therapy might also be useful [10,28]. However, the percentage of patients actually receiving treatment is probably small [13,29]. Patients might visit a hospital and might receive a diagnosis of ALD only after symptoms such as jaundice appear. Some patients are resistant to intervention by psychiatric and multidisciplinary teams because they are not properly diagnosed with AUD or because they do not accept a diagnosis of AUD.

Biomarkers are convenient for assessing alcohol consumption in an outpatient setting [30,31]. Routinely available laboratory tests such as mean corpuscular volume and GGT can be done easily, but they do not show greater amounts of alcohol consumption directly. Blood-urine alcohol concentration has a short half-life. For that reason, it might not reflect the amount of alcohol consumed during outpatient evaluations. The %CDT has been used as a biomarker to detect heavy (>50 g) alcohol consumption within the prior several weeks [10,30-32]. Suzuki et al [32] reported a cut-off value for excessive drinkers (men ≥60 g/day, women ≥40 g/day) of 1.9% (sensitivity=0.77, specificity=0.77). Reportedly, the combination of GGT and %CDT (GGT-CDT) shows higher sensitivity for detection of heavy drinking and higher prediction accuracy for detection of excessive alcohol consumption [27,33]. Hietala et al [27] reported the cut-off value for heavy drinkers as 4.18 for men and as 3.81 for women using a derived formula (sensitivity=0.90, specificity=0.98). However, the use of %CDT for patients with liver disease remains limited because of its low sensitivity and low specificity [30]. Elevated %CDT values have been noted frequently in liver transplant candidates with end-stage liver disease [34]. Currently, other biomarkers such as ethyl glucuronide and phosphatidylethanol are used widely [35].

For this study, liver function and %CDT were evaluated because other alcohol consumption–specific markers have not been commercially available. Although the number of patients was too low to evaluate the diagnostic performance of %CDT, this measure can reflect changes related to alcohol consumption for time series monitoring of individual cases. However, we found no correlation between self-reported ethanol consumption and %CDT. The sensitivity of %CDT was low probably because of the large number of patients with advanced cirrhosis.

The usefulness of diaries for recording alcohol consumption was demonstrated, but their potential as an adjunctive test for judging treatment effects was also suggested by our results.

Nevertheless, the tendencies found in diary entries suggest that input rates were high among abstinent patients, whereas the rates were low among continuing drinkers, also indicating a long lag separating the date of entry and the date of diary completion. Considering the resistance of patients to having their drinking habits known by their treating physicians, some patients might refuse direct testing for alcohol consumption as well.

One-half of the patients who completed the study achieved 8 weeks of self-reported abstinence. By checking the physician's view of the server app, the daily drinking status can be noted at a glance. The alcohol consumption amount was graphed, making it possible to ascertain the self-reported amount of alcohol consumed. Most patients in this study had liver cirrhosis. Of the 5 patients who abstained from alcohol, 4 had severe Child class C cirrhosis, suggesting that more severe conditions might be a factor facilitating abstinence. Other factors such as the absence of a comorbid psychiatric disorder and having a job were also notable.

For a short period of time, a trend toward improved nutritional status (albumin) was apparent as a result of abstinence by patients using the smartphone journaling app. Other indicators were not found to be significant in this study. However, the fact that patients' proactive approaches to abstinence behavior engender improved liver function can motivate them to seek and benefit from future treatment.

One benefit of using a smartphone app is the possibility of identifying when the patient entered a record. For paper-based diaries, it is difficult to ascertain when the patient makes an entry. In this study, the results of analyzing input record date lags in the data indicated that patients who were sober tended to input data into the smartphone journaling app almost every day. By contrast, nonabstaining patients tended to enter many records together at one time, according to the date of their medical visit. In such cases, the input record date lags tended to be greater in the fourth and eighth weeks of the study. Based on these considerations, a certain number of patients probably used the smartphone journaling app with some awareness of the date of their medical visit. Therefore, providing automatic feedback through the smartphone app is necessary to raise awareness and motivation. Alternatively, an input time lag might provide a hint that a patient needs more care.

### Limitations

This study has several limitations. The smartphone journaling app used for this study was set up so that a one-line diary could not be saved without entering a single word, irrespective of whether the user had been drinking. Results from a questionnaire completed after using the app indicated that “entering daily diary entries is difficult.” Possible future improvements to the app described herein include allowing free-form diary entries and allowing a stamp function that can help users simply express their mood on a given day.

Another limitation is this current study had no control arm participants who had received treatment (4 weeks and 8 weeks) without the smartphone app. We assume that such control cases would show no significant change in the short term (4 weeks or 8 weeks). However, a larger scale and double-arm, randomized controlled trial is highly desirable.

Nonetheless, this pilot study showed positive results among patients with ALD who were recommended to reduce or quit drinking. Among multiple functions provided by the smartphone app (journaling, physician’s view-based feedback), precisely which function was the main trigger demands further study.

### Conclusions

In conclusion, the use of an alcohol use journaling app in combination with hepatologists’ follow-up was associated with reduction of daily alcohol intake and improvement of liver function test results. The positional gap separating patients and physicians was narrowed by sharing the journaling app. Further studies are expected to elucidate the use of digital tools in an ambulatory care setting.

## Appendix

Multimedia Appendix 1 Input-record date lag, daily alcohol use, and date of hospital visit. (a) and (b) represent cases without complete sobriety; (c) and (d) represent cases with complete sobriety.

Multimedia Appendix 2 Questionnaire about a journaling smartphone-app.

## Abbreviations

A-CHESS: Addiction-Comprehensive Health Enhancement Support System
ALD: alcohol-related liver disease
AUD: alcohol use disorder
AUDIT: Alcohol Use Disorders Inventory Test
CDT: carbohydrate-deficient transferrin
GGT: gamma glutamyl transferase

## Data Availability

The data sets analyzed during the current study are not publicly available for reasons of patient privacy, but they are available from the corresponding author on reasonable request.
