# Supplementary figures and images for: Narrowing the Patient–Physician Gap Based on Self-Reporting and Monthly Hepatologist Feedback for Patients With Alcohol-Related Liver Disease: Interventional Pilot Study Using a Journaling Smartphone App
Source: JMIR Form Res. 2023 Dec 19;7:e44762. doi: 10.2196/44762 (PMC10762609; doi:10.2196/44762)

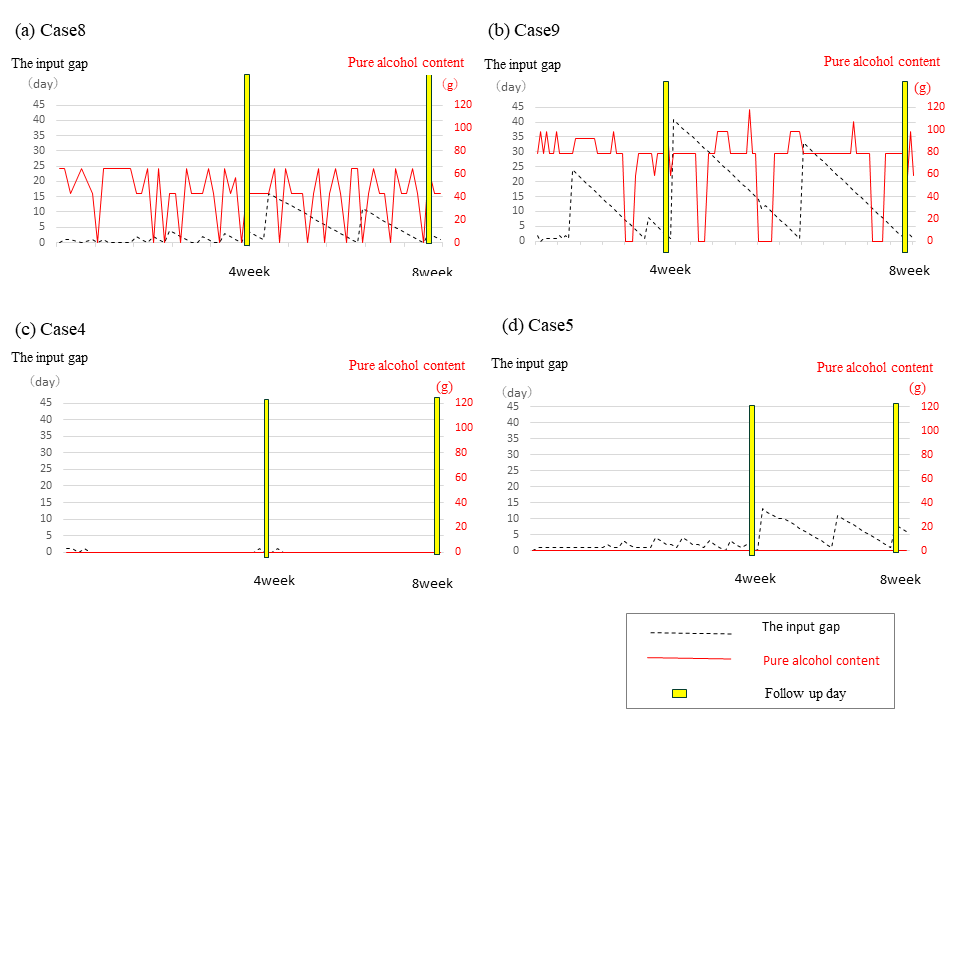

Supplement: Multimedia Appendix 1 [file formative_v7i1e44762_app1.png]

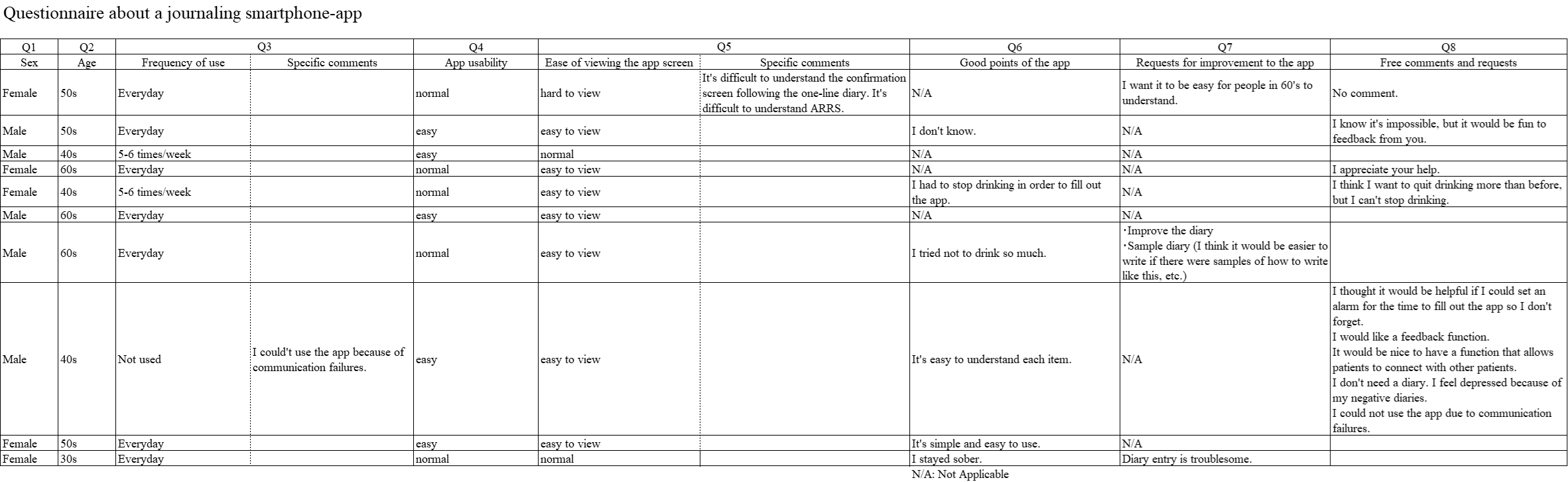

Supplement: Multimedia Appendix 2 [file formative_v7i1e44762_app2.png]
